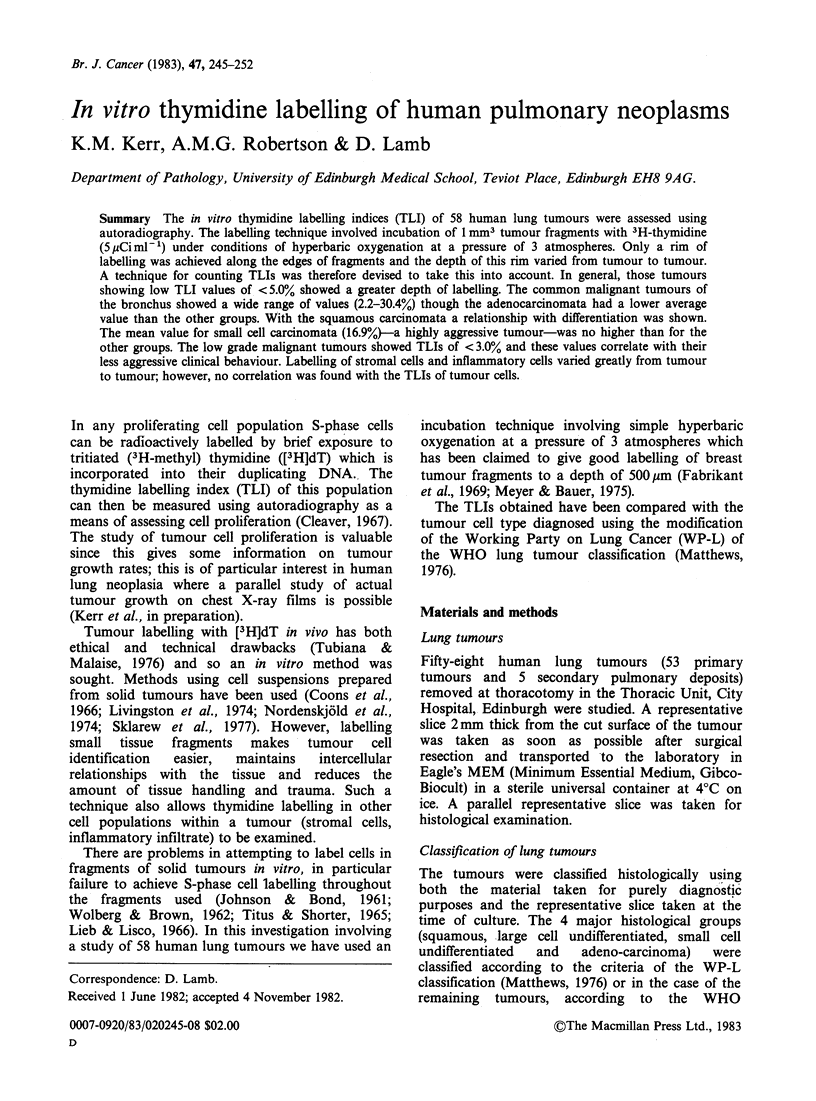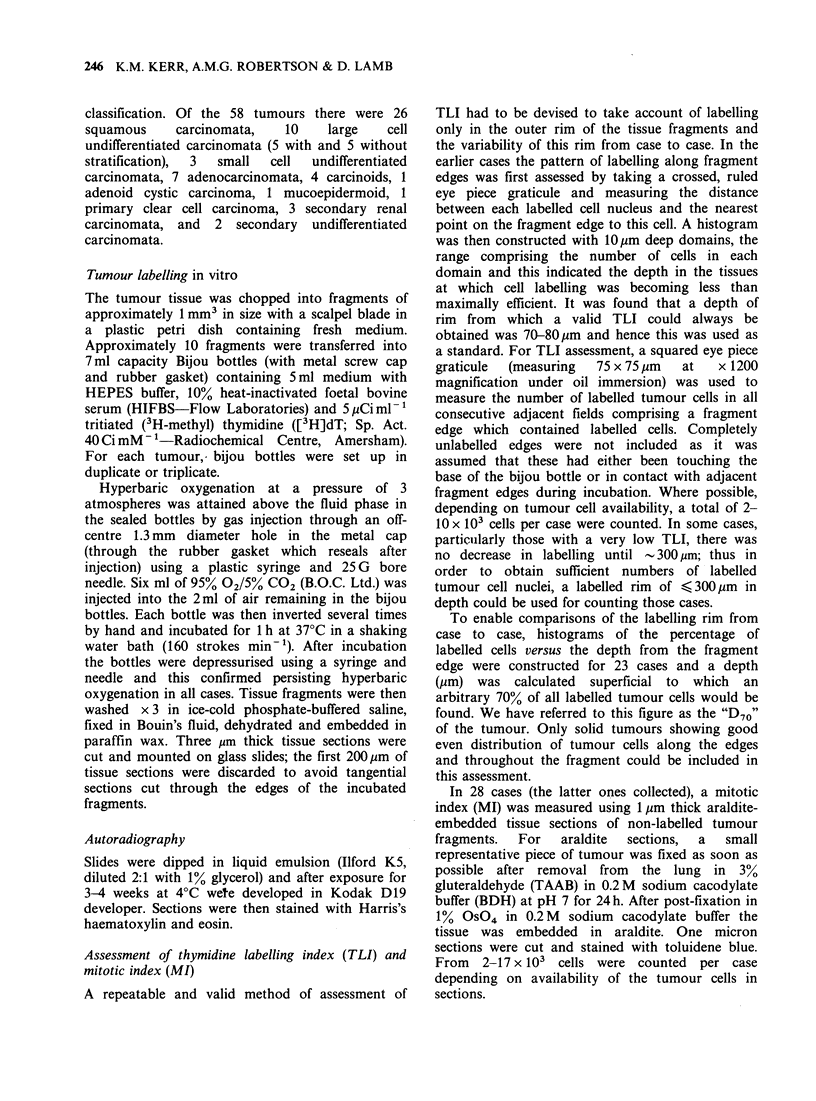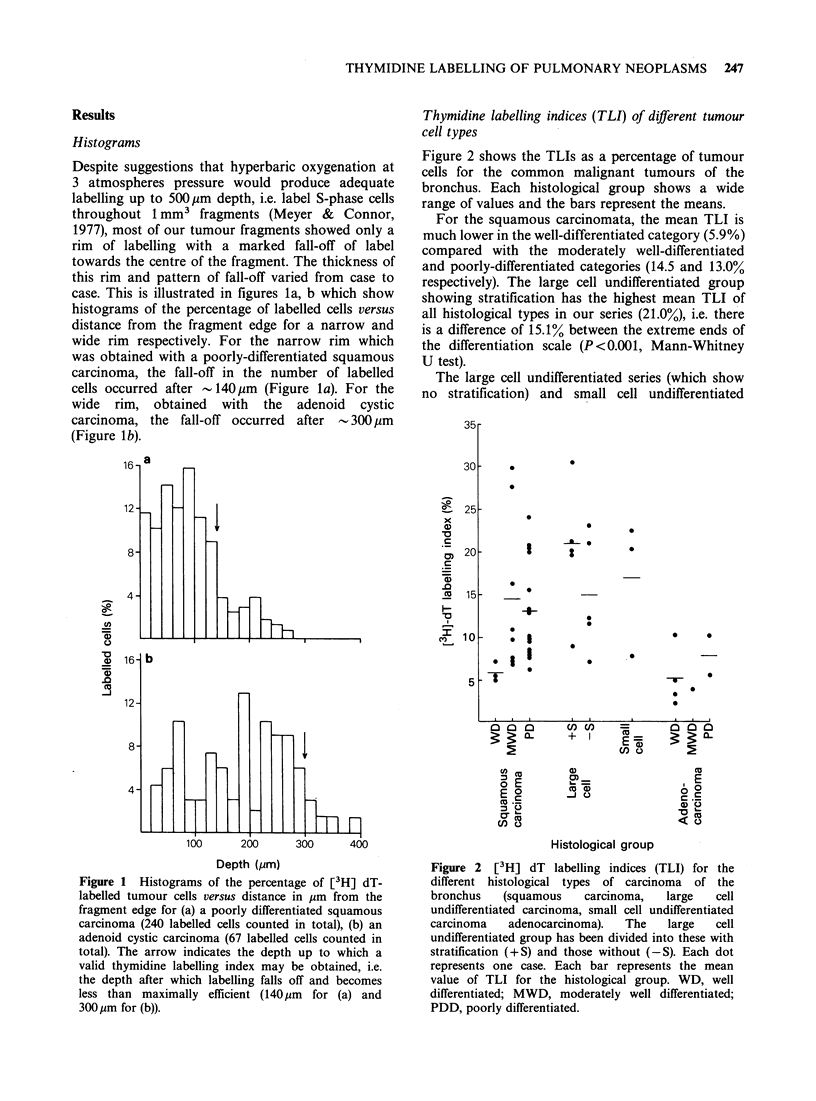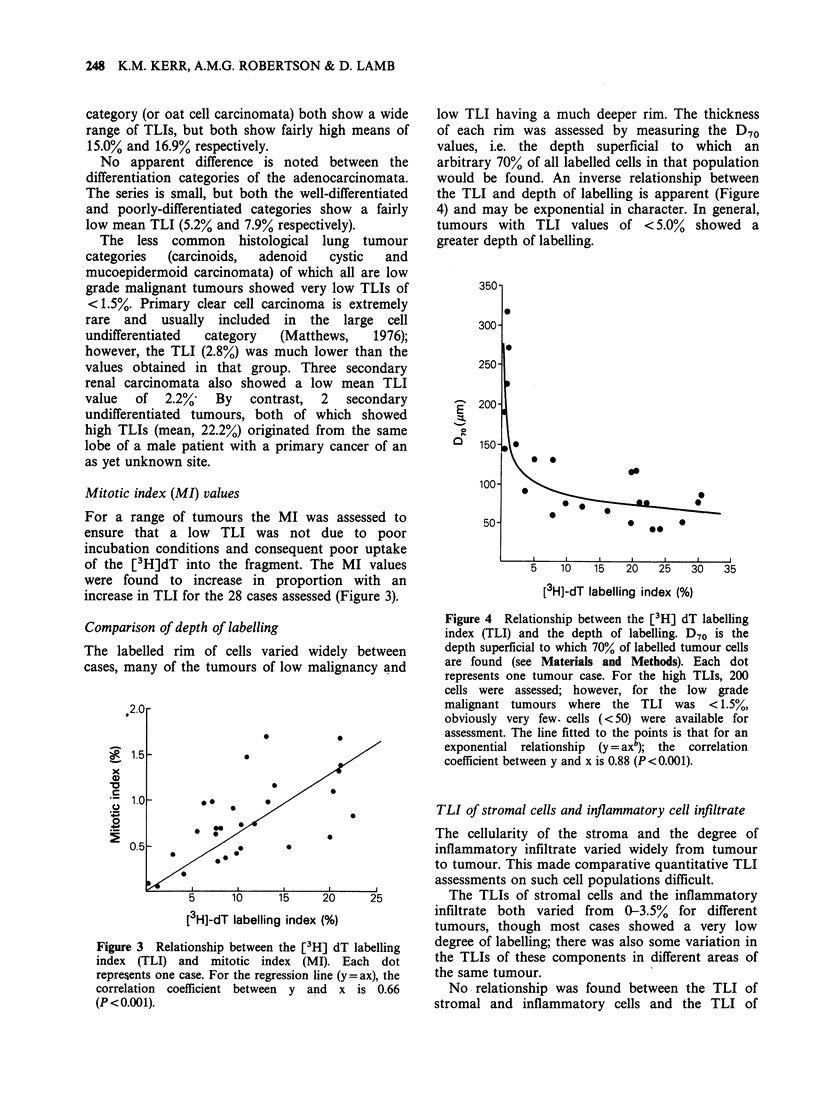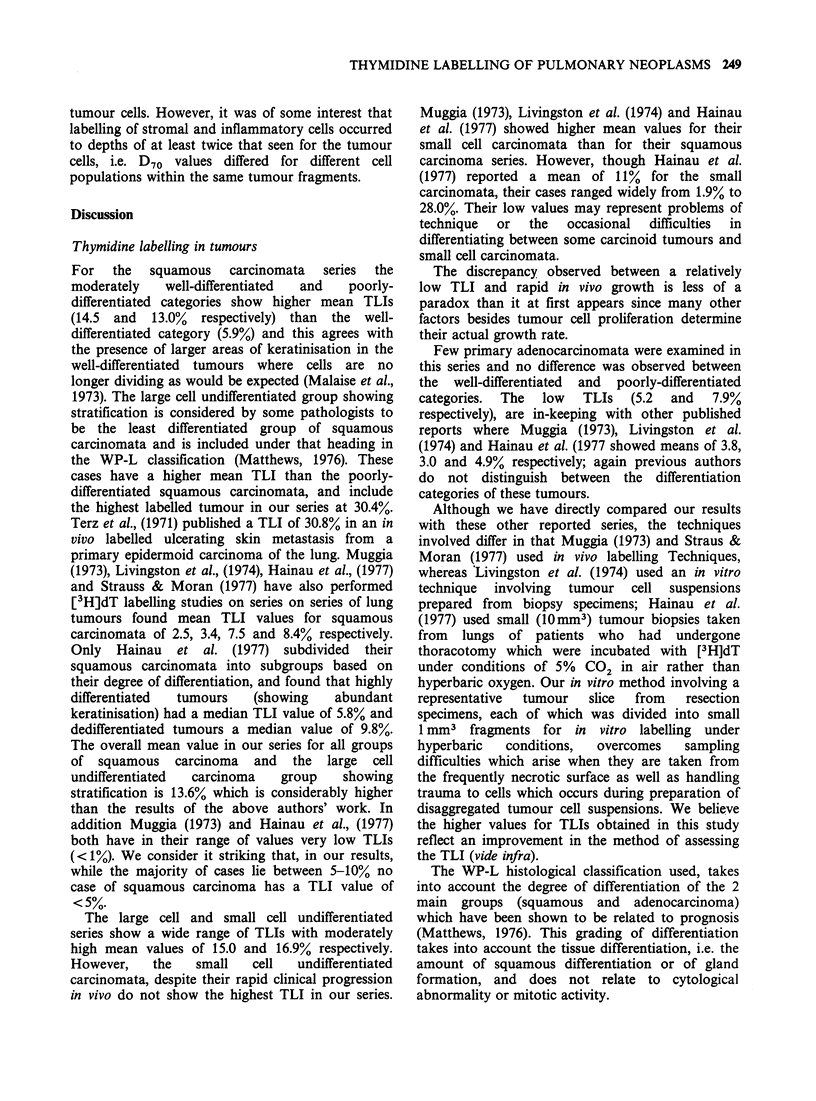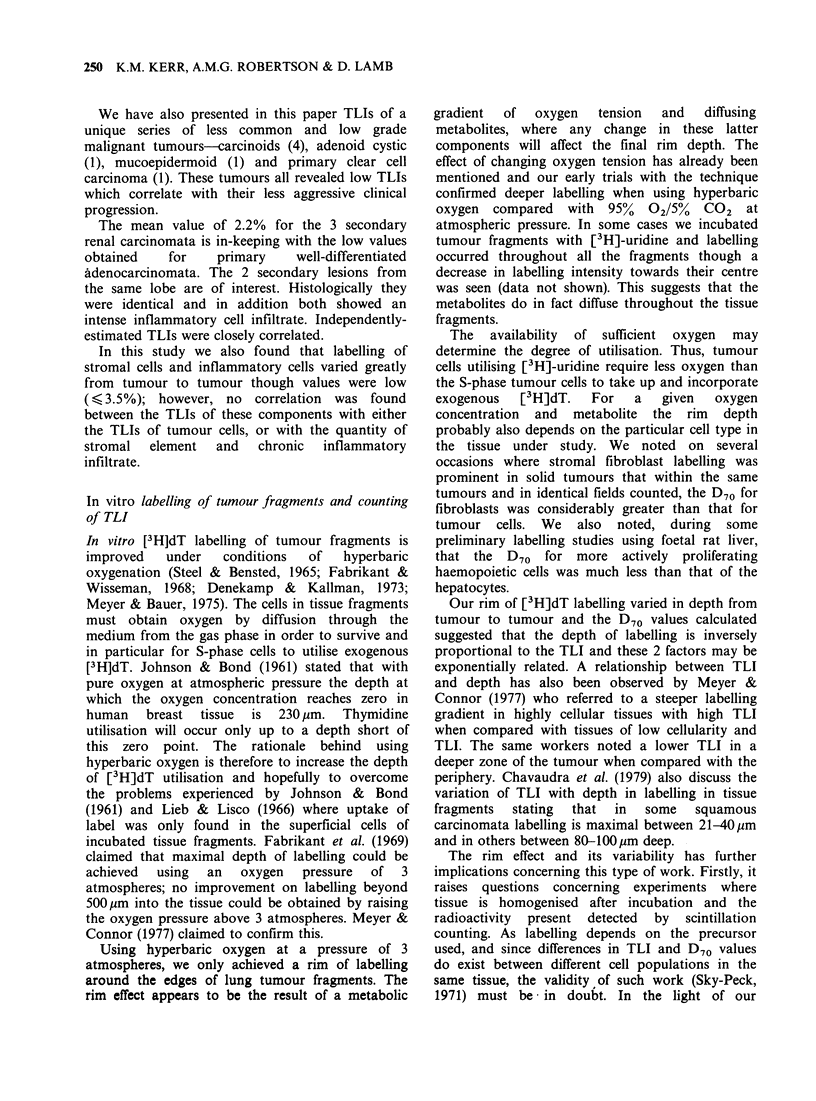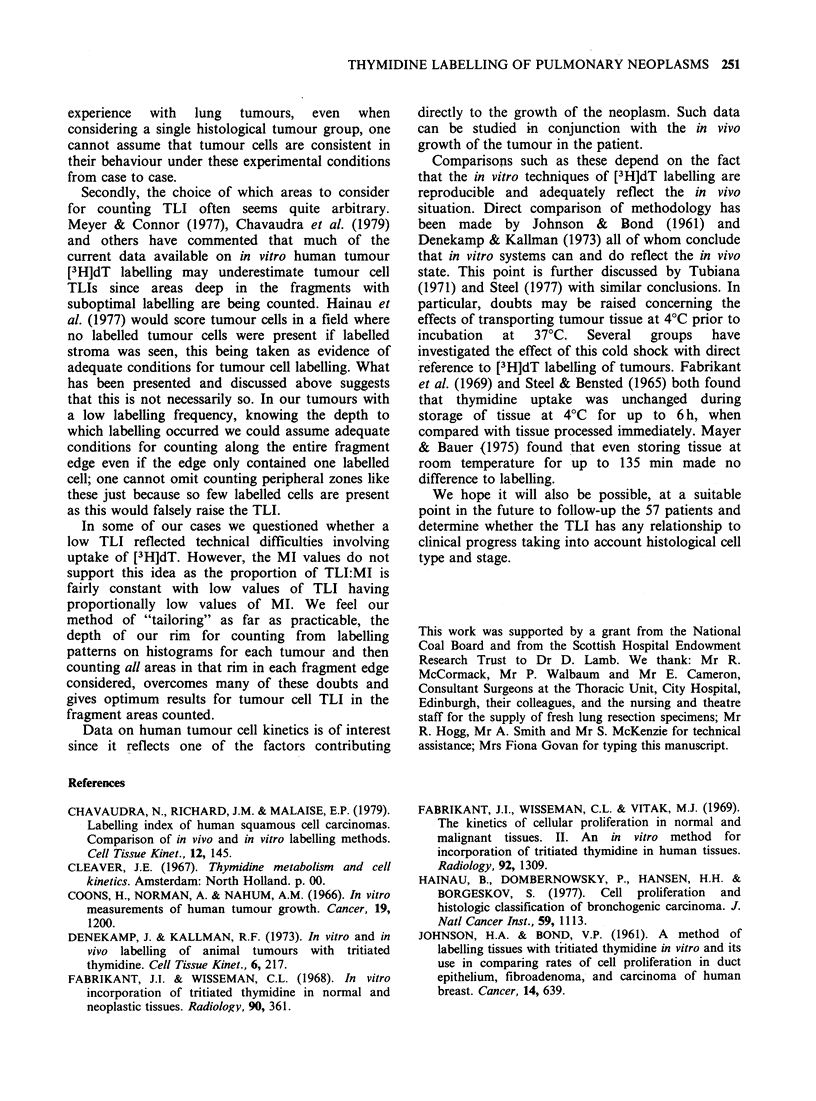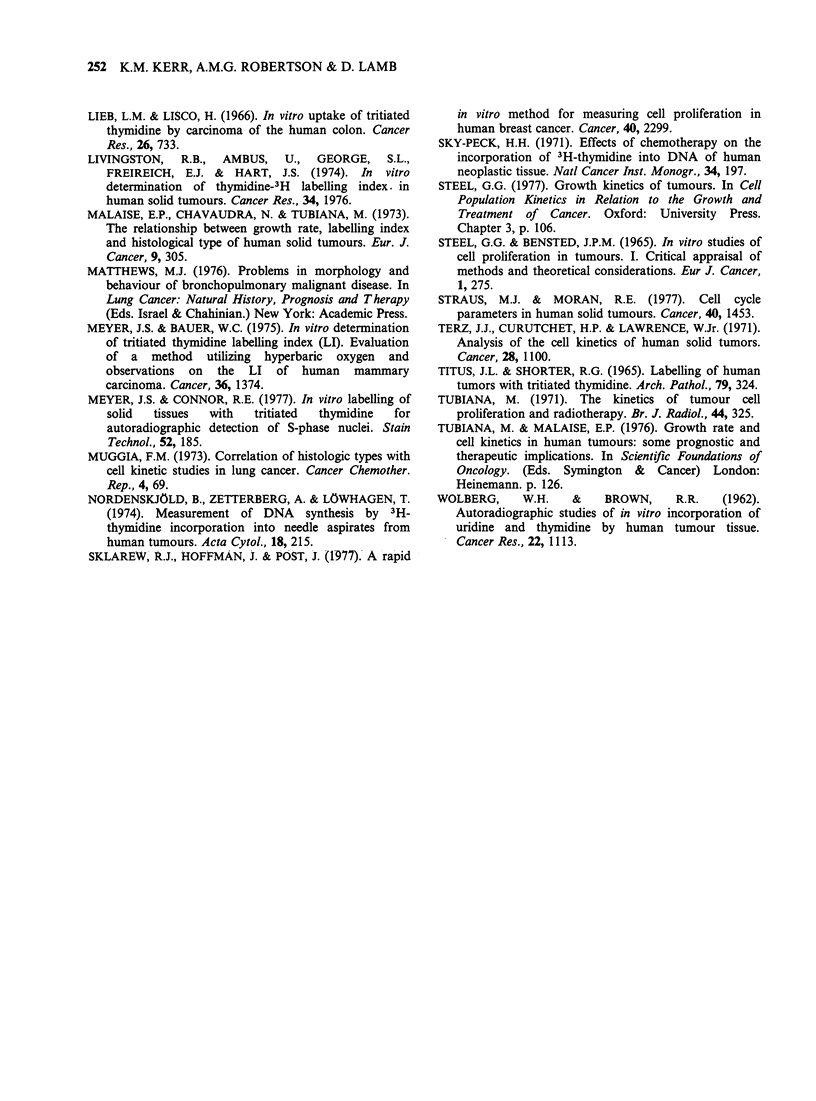# In vitro thymidine labelling of human pulmonary neoplasms.

**DOI:** 10.1038/bjc.1983.32

**Published:** 1983-02

**Authors:** K. M. Kerr, A. M. Robertson, D. Lamb

## Abstract

The in vitro thymidine labelling indices (TLI) of 58 human lung tumours were assessed using autoradiography. The labelling technique involved incubation of 1 mm3 tumour fragments with 3H-thymidine (5 muCi ml-1) under conditions of hyperbaric oxygenation at a pressure of 3 atmospheres. Only a rim of labelling was achieved along the edges of fragments and the depth of this rim varied from tumour to tumour. A technique for counting TLIs was therefore devised to take this into account. In general, those tumours showing low TLI values of less than 5.0% showed a greater depth of labelling. The common malignant tumours of the bronchus showed a wide range of values (2.2-30.4%) though the adenocarcinomata had a lower average value than the other groups. With the squamous carcinomata a relationship with differentiation was shown. The mean value for small cell carcinomata (16.9%)--a highly aggressive tumour--was no higher than for the other groups. The low grade malignant tumours showed TLIs of less than 3.0% and these values correlate with their less aggressive clinical behaviour. Labelling of stromal cells and inflammatory cells varied greatly from tumour to tumour; however, no correlation was found with the TLIs of tumour cells.


*Br. J. Cancer* (1983), **47**, 245–252

### *In vitro* thymidine labelling of human pulmonary neoplasms

K.M. Kerr, A.M.G. Robertson & D. Lamb

*Department of Pathology, University of Edinburgh Medical School, Teviot Place, Edinburgh EH8 9AG.*

**Summary** The *in vitro* thymidine labelling indices (TLI) of 58 human lung tumours were assessed using autoradiography. The labelling technique involved incubation of 1 $mm^3$ tumour fragments with $^3$H-thymidine (5 $\mu Ci\, ml^{-1}$) under conditions of hyperbaric oxygenation at a pressure of 3 atmospheres. Only a rim of labelling was achieved along the edges of fragments and the depth of this rim varied from tumour to tumour. A technique for counting TLIs was therefore devised to take this into account. In general, those tumours showing low TLI values of <5.0% showed a greater depth of labelling. The common malignant tumours of the bronchus showed a wide range of values (2.2–30.4%) though the adenocarcinomata had a lower average value than the other groups. With the squamous carcinomata a relationship with differentiation was shown. The mean value for small cell carcinomata (16.9%)—a highly aggressive tumour—was no higher than for the other groups. The low grade malignant tumours showed TLIs of <3.0% and these values correlate with their less aggressive clinical behaviour. Labelling of stromal cells and inflammatory cells varied greatly from tumour to tumour; however, no correlation was found with the TLIs of tumour cells.

In any proliferating cell population S-phase cells can be radioactively labelled by brief exposure to tritiated ($^3$H-methyl) thymidine ([$^3$H]dT) which is incorporated into their duplicating DNA. The thymidine labelling index (TLI) of this population can then be measured using autoradiography as a means of assessing cell proliferation (Cleaver, 1967). The study of tumour cell proliferation is valuable since this gives some information on tumour growth rates; this is of particular interest in human lung neoplasia where a parallel study of actual tumour growth on chest X-ray films is possible (Kerr *et al.*, in preparation).

Tumour labelling with [$^3$H]dT *in vivo* has both ethical and technical drawbacks (Tubiana & Malaise, 1976) and so an *in vitro* method was sought. Methods using cell suspensions prepared from solid tumours have been used (Coons *et al.*, 1966; Livingston *et al.*, 1974; Nordenskjöld *et al.*, 1974; Sklarew *et al.*, 1977). However, labelling small tissue fragments makes tumour cell identification easier, maintains intercellular relationships with the tissue and reduces the amount of tissue handling and trauma. Such a technique also allows thymidine labelling in other cell populations within a tumour (stromal cells, inflammatory infiltrate) to be examined.

There are problems in attempting to label cells in fragments of solid tumours *in vitro*, in particular failure to achieve S-phase cell labelling throughout the fragments used (Johnson & Bond, 1961; Wolberg & Brown, 1962; Titus & Shorter, 1965; Lieb & Lisco, 1966). In this investigation involving a study of 58 human lung tumours we have used an incubation technique involving simple hyperbaric oxygenation at a pressure of 3 atmospheres which has been claimed to give good labelling of breast tumour fragments to a depth of 500 $\mu$m (Fabrikant *et al.*, 1969; Meyer & Bauer, 1975).

The TLIs obtained have been compared with the tumour cell type diagnosed using the modification of the Working Party on Lung Cancer (WP-L) of the WHO lung tumour classification (Matthews, 1976).

### Materials and methods

### *Lung tumours*

Fifty-eight human lung tumours (53 primary tumours and 5 secondary pulmonary deposits) removed at thoracotomy in the Thoracic Unit, City Hospital, Edinburgh were studied. A representative slice 2 mm thick from the cut surface of the tumour was taken as soon as possible after surgical resection and transported to the laboratory in Eagle's MEM (Minimum Essential Medium, Gibco-Biocult) in a sterile universal container at 4°C on ice. A parallel representative slice was taken for histological examination.

### *Classification of lung tumours*

The tumours were classified histologically using both the material taken for purely diagnostic purposes and the representative slice taken at the time of culture. The 4 major histological groups (squamous, large cell undifferentiated, small cell undifferentiated and adeno-carcinoma) were classified according to the criteria of the WP-L classification (Matthews, 1976) or in the case of the remaining tumours, according to the WHO

Correspondence: D. Lamb.

Received 1 June 1982; accepted 4 November 1982.

0007-0920/83/020245-08 $02.00

©The Macmillan Press Ltd., 1983

classification. Of the 58 tumours there were 26 squamous carcinomata, 10 large cell undifferentiated carcinomata (5 with and 5 without stratification), 3 small cell undifferentiated carcinomata, 7 adenocarcinomata, 4 carcinoids, 1 adenoid cystic carcinoma, 1 mucoepidermoid, 1 primary clear cell carcinoma, 3 secondary renal carcinomata, and 2 secondary undifferentiated carcinomata.

*Tumour labelling* in vitro

The tumour tissue was chopped into fragments of approximately 1 $mm^3$ in size with a scalpel blade in a plastic petri dish containing fresh medium. Approximately 10 fragments were transferred into 7 ml capacity Bijou bottles (with metal screw cap and rubber gasket) containing 5 ml medium with HEPES buffer, 10% heat-inactivated foetal bovine serum (HIFBS—Flow Laboratories) and 5 $\mu Ci\,ml^{-1}$ tritiated ($^3$H-methyl) thymidine ([$^3$H]dT; Sp. Act. 40 Ci $mM^{-1}$—Radiochemical Centre, Amersham). For each tumour, bijou bottles were set up in duplicate or triplicate.

Hyperbaric oxygenation at a pressure of 3 atmospheres was attained above the fluid phase in the sealed bottles by gas injection through an off-centre 1.3 mm diameter hole in the metal cap (through the rubber gasket which reseals after injection) using a plastic syringe and 25 G bore needle. Six ml of 95% $O_2$/5% $CO_2$ (B.O.C. Ltd.) was injected into the 2 ml of air remaining in the bijou bottles. Each bottle was then inverted several times by hand and incubated for 1 h at 37°C in a shaking water bath (160 strokes $min^{-1}$). After incubation the bottles were depressurised using a syringe and needle and this confirmed persisting hyperbaric oxygenation in all cases. Tissue fragments were then washed ×3 in ice-cold phosphate-buffered saline, fixed in Bouin's fluid, dehydrated and embedded in paraffin wax. Three μm thick tissue sections were cut and mounted on glass slides; the first 200 μm of tissue sections were discarded to avoid tangential sections cut through the edges of the incubated fragments.

*Autoradiography*

Slides were dipped in liquid emulsion (Ilford K5, diluted 2:1 with 1% glycerol) and after exposure for 3–4 weeks at 4°C were developed in Kodak D19 developer. Sections were then stained with Harris's haematoxylin and eosin.

*Assessment of thymidine labelling index (TLI) and mitotic index (MI)*

A repeatable and valid method of assessment of TLI had to be devised to take account of labelling only in the outer rim of the tissue fragments and the variability of this rim from case to case. In the earlier cases the pattern of labelling along fragment edges was first assessed by taking a crossed, ruled eye piece graticule and measuring the distance between each labelled cell nucleus and the nearest point on the fragment edge to this cell. A histogram was then constructed with 10 μm deep domains, the range comprising the number of cells in each domain and this indicated the depth in the tissues at which cell labelling was becoming less than maximally efficient. It was found that a depth of rim from which a valid TLI could always be obtained was 70–80 μm and hence this was used as a standard. For TLI assessment, a squared eye piece graticule (measuring 75 × 75 μm at ×1200 magnification under oil immersion) was used to measure the number of labelled tumour cells in all consecutive adjacent fields comprising a fragment edge which contained labelled cells. Completely unlabelled edges were not included as it was assumed that these had either been touching the base of the bijou bottle or in contact with adjacent fragment edges during incubation. Where possible, depending on tumour cell availability, a total of 2–10 × $10^3$ cells per case were counted. In some cases, particularly those with a very low TLI, there was no decrease in labelling until ~300 μm; thus in order to obtain sufficient numbers of labelled tumour cell nuclei, a labelled rim of ⩽300 μm in depth could be used for counting those cases.

To enable comparisons of the labelling rim from case to case, histograms of the percentage of labelled cells *versus* the depth from the fragment edge were constructed for 23 cases and a depth (μm) was calculated superficial to which an arbitrary 70% of all labelled tumour cells would be found. We have referred to this figure as the "$D_{70}$" of the tumour. Only solid tumours showing good even distribution of tumour cells along the edges and throughout the fragment could be included in this assessment.

In 28 cases (the latter ones collected), a mitotic index (MI) was measured using 1 μm thick araldite-embedded tissue sections of non-labelled tumour fragments. For araldite sections, a small representative piece of tumour was fixed as soon as possible after removal from the lung in 3% gluteraldehyde (TAAB) in 0.2 M sodium cacodylate buffer (BDH) at pH 7 for 24 h. After post-fixation in 1% $OsO_4$ in 0.2 M sodium cacodylate buffer the tissue was embedded in araldite. One micron sections were cut and stained with toluidene blue. From 2–17 × $10^3$ cells were counted per case depending on availability of the tumour cells in sections.

### Results

### *Histograms*

Despite suggestions that hyperbaric oxygenation at 3 atmospheres pressure would produce adequate labelling up to 500 μm depth, i.e. label S-phase cells throughout 1 mm$^3$ fragments (Meyer & Connor, 1977), most of our tumour fragments showed only a rim of labelling with a marked fall-off of label towards the centre of the fragment. The thickness of this rim and pattern of fall-off varied from case to case. This is illustrated in figures 1a, b which show histograms of the percentage of labelled cells *versus* distance from the fragment edge for a narrow and wide rim respectively. For the narrow rim which was obtained with a poorly-differentiated squamous carcinoma, the fall-off in the number of labelled cells occurred after ~140 μm (Figure 1*a*). For the wide rim, obtained with the adenoid cystic carcinoma, the fall-off occurred after ~300 μm (Figure 1*b*).

**Figure 1** Histograms of the percentage of [$^3$H] dT-labelled tumour cells *versus* distance in μm from the fragment edge for (a) a poorly differentiated squamous carcinoma (240 labelled cells counted in total), (b) an adenoid cystic carcinoma (67 labelled cells counted in total). The arrow indicates the depth up to which a valid thymidine labelling index may be obtained, i.e. the depth after which labelling falls off and becomes less than maximally efficient (140 μm for (a) and 300 μm for (b)).

### *Thymidine labelling indices (TLI) of different tumour cell types*

Figure 2 shows the TLIs as a percentage of tumour cells for the common malignant tumours of the bronchus. Each histological group shows a wide range of values and the bars represent the means.

For the squamous carcinomata, the mean TLI is much lower in the well-differentiated category (5.9%) compared with the moderately well-differentiated and poorly-differentiated categories (14.5 and 13.0% respectively). The large cell undifferentiated group showing stratification has the highest mean TLI of all histological types in our series (21.0%), i.e. there is a difference of 15.1% between the extreme ends of the differentiation scale ($P<0.001$, Mann-Whitney U test).

The large cell undifferentiated series (which show no stratification) and small cell undifferentiated

**Figure 2** [$^3$H] dT labelling indices (TLI) for the different histological types of carcinoma of the bronchus (squamous carcinoma, large cell undifferentiated carcinoma, small cell undifferentiated carcinoma adenocarcinoma). The large cell undifferentiated group has been divided into these with stratification (+S) and those without (−S). Each dot represents one case. Each bar represents the mean value of TLI for the histological group. WD, well differentiated; MWD, moderately well differentiated; PDD, poorly differentiated.

category (or oat cell carcinomata) both show a wide range of TLIs, but both show fairly high means of 15.0% and 16.9% respectively.

No apparent difference is noted between the differentiation categories of the adenocarcinomata. The series is small, but both the well-differentiated and poorly-differentiated categories show a fairly low mean TLI (5.2% and 7.9% respectively).

The less common histological lung tumour categories (carcinoids, adenoid cystic and mucoepidermoid carcinomata) of which all are low grade malignant tumours showed very low TLIs of <1.5%. Primary clear cell carcinoma is extremely rare and usually included in the large cell undifferentiated category (Matthews, 1976); however, the TLI (2.8%) was much lower than the values obtained in that group. Three secondary renal carcinomata also showed a low mean TLI value of 2.2%· By contrast, 2 secondary undifferentiated tumours, both of which showed high TLIs (mean, 22.2%) originated from the same lobe of a male patient with a primary cancer of an as yet unknown site.

*Mitotic index (MI) values*

For a range of tumours the MI was assessed to ensure that a low TLI was not due to poor incubation conditions and consequent poor uptake of the [$^3$H]dT into the fragment. The MI values were found to increase in proportion with an increase in TLI for the 28 cases assessed (Figure 3).

*Comparison of depth of labelling*

The labelled rim of cells varied widely between cases, many of the tumours of low malignancy and low TLI having a much deeper rim. The thickness of each rim was assessed by measuring the $D_{70}$ values, i.e. the depth superficial to which an arbitrary 70% of all labelled cells in that population would be found. An inverse relationship between the TLI and depth of labelling is apparent (Figure 4) and may be exponential in character. In general, tumours with TLI values of <5.0% showed a greater depth of labelling.

**Figure 3** Relationship between the [$^3$H] dT labelling index (TLI) and mitotic index (MI). Each dot represents one case. For the regression line (y = ax), the correlation coefficient between y and x is 0.66 ($P<0.001$).

**Figure 4** Relationship between the [$^3$H] dT labelling index (TLI) and the depth of labelling. $D_{70}$ is the depth superficial to which 70% of labelled tumour cells are found (see **Materials and Methods**). Each dot represents one tumour case. For the high TLIs, 200 cells were assessed; however, for the low grade malignant tumours where the TLI was <1.5%, obviously very few cells (<50) were available for assessment. The line fitted to the points is that for an exponential relationship ($y=ax^b$); the correlation coefficient between y and x is 0.88 ($P<0.001$).

*TLI of stromal cells and inflammatory cell infiltrate*

The cellularity of the stroma and the degree of inflammatory infiltrate varied widely from tumour to tumour. This made comparative quantitative TLI assessments on such cell populations difficult.

The TLIs of stromal cells and the inflammatory infiltrate both varied from 0–3.5% for different tumours, though most cases showed a very low degree of labelling; there was also some variation in the TLIs of these components in different areas of the same tumour.

No relationship was found between the TLI of stromal and inflammatory cells and the TLI of tumour cells. However, it was of some interest that labelling of stromal and inflammatory cells occurred to depths of at least twice that seen for the tumour cells, i.e. $D_{70}$ values differed for different cell populations within the same tumour fragments.

### Discussion

*Thymidine labelling in tumours*

For the squamous carcinomata series the moderately well-differentiated and poorly-differentiated categories show higher mean TLIs (14.5 and 13.0% respectively) than the well-differentiated category (5.9%) and this agrees with the presence of larger areas of keratinisation in the well-differentiated tumours where cells are no longer dividing as would be expected (Malaise *et al.*, 1973). The large cell undifferentiated group showing stratification is considered by some pathologists to be the least differentiated group of squamous carcinomata and is included under that heading in the WP-L classification (Matthews, 1976). These cases have a higher mean TLI than the poorly-differentiated squamous carcinomata, and include the highest labelled tumour in our series at 30.4%. Terz *et al.*, (1971) published a TLI of 30.8% in an *in vivo* labelled ulcerating skin metastasis from a primary epidermoid carcinoma of the lung. Muggia (1973), Livingston *et al.*, (1974), Hainau *et al.*, (1977) and Strauss & Moran (1977) have also performed [$^3$H]dT labelling studies on series on series of lung tumours found mean TLI values for squamous carcinomata of 2.5, 3.4, 7.5 and 8.4% respectively. Only Hainau *et al.* (1977) subdivided their squamous carcinomata into subgroups based on their degree of differentiation, and found that highly differentiated tumours (showing abundant keratinisation) had a median TLI value of 5.8% and dedifferentiated tumours a median value of 9.8%. The overall mean value in our series for all groups of squamous carcinoma and the large cell undifferentiated carcinoma group showing stratification is 13.6% which is considerably higher than the results of the above authors' work. In addition Muggia (1973) and Hainau *et al.*, (1977) both have in their range of values very low TLIs ($<1$%). We consider it striking that, in our results, while the majority of cases lie between 5–10% no case of squamous carcinoma has a TLI value of $<5$%.

The large cell and small cell undifferentiated series show a wide range of TLIs with moderately high mean values of 15.0 and 16.9% respectively. However, the small cell undifferentiated carcinomata, despite their rapid clinical progression *in vivo* do not show the highest TLI in our series. Muggia (1973), Livingston *et al.* (1974) and Hainau *et al.* (1977) showed higher mean values for their small cell carcinomata than for their squamous carcinoma series. However, though Hainau *et al.* (1977) reported a mean of 11% for the small carcinomata, their cases ranged widely from 1.9% to 28.0%. Their low values may represent problems of technique or the occasional difficulties in differentiating between some carcinoid tumours and small cell carcinomata.

The discrepancy observed between a relatively low TLI and rapid *in vivo* growth is less of a paradox than it at first appears since many other factors besides tumour cell proliferation determine their actual growth rate.

Few primary adenocarcinomata were examined in this series and no difference was observed between the well-differentiated and poorly-differentiated categories. The low TLIs (5.2 and 7.9% respectively), are in-keeping with other published reports where Muggia (1973), Livingston *et al.* (1974) and Hainau *et al.* (1977 showed means of 3.8, 3.0 and 4.9% respectively; again previous authors do not distinguish between the differentiation categories of these tumours.

Although we have directly compared our results with these other reported series, the techniques involved differ in that Muggia (1973) and Straus & Moran (1977) used *in vivo* labelling Techniques, whereas Livingston *et al.* (1974) used an *in vitro* technique involving tumour cell suspensions prepared from biopsy specimens; Hainau *et al.* (1977) used small (10 mm$^3$) tumour biopsies taken from lungs of patients who had undergone thoracotomy which were incubated with [$^3$H]dT under conditions of 5% $CO_2$ in air rather than hyperbaric oxygen. Our *in vitro* method involving a representative tumour slice from resection specimens, each of which was divided into small 1 mm$^3$ fragments for *in vitro* labelling under hyperbaric conditions, overcomes sampling difficulties which arise when they are taken from the frequently necrotic surface as well as handling trauma to cells which occurs during preparation of disaggregated tumour cell suspensions. We believe the higher values for TLIs obtained in this study reflect an improvement in the method of assessing the TLI (*vide infra*).

The WP-L histological classification used, takes into account the degree of differentiation of the 2 main groups (squamous and adenocarcinoma) which have been shown to be related to prognosis (Matthews, 1976). This grading of differentiation takes into account the tissue differentiation, i.e. the amount of squamous differentiation or of gland formation, and does not relate to cytological abnormality or mitotic activity.

We have also presented in this paper TLIs of a unique series of less common and low grade malignant tumours—carcinoids (4), adenoid cystic (1), mucoepidermoid (1) and primary clear cell carcinoma (1). These tumours all revealed low TLIs which correlate with their less aggressive clinical progression.

The mean value of 2.2% for the 3 secondary renal carcinomata is in-keeping with the low values obtained for primary well-differentiated adenocarcinomata. The 2 secondary lesions from the same lobe are of interest. Histologically they were identical and in addition both showed an intense inflammatory cell infiltrate. Independently-estimated TLIs were closely correlated.

In this study we also found that labelling of stromal cells and inflammatory cells varied greatly from tumour to tumour though values were low ($\leqslant$3.5%); however, no correlation was found between the TLIs of these components with either the TLIs of tumour cells, or with the quantity of stromal element and chronic inflammatory infiltrate.

### In vitro *labelling of tumour fragments and counting of TLI*

*In vitro* [$^3$H]dT labelling of tumour fragments is improved under conditions of hyperbaric oxygenation (Steel & Bensted, 1965; Fabrikant & Wisseman, 1968; Denekamp & Kallman, 1973; Meyer & Bauer, 1975). The cells in tissue fragments must obtain oxygen by diffusion through the medium from the gas phase in order to survive and in particular for S-phase cells to utilise exogenous [$^3$H]dT. Johnson & Bond (1961) stated that with pure oxygen at atmospheric pressure the depth at which the oxygen concentration reaches zero in human breast tissue is 230 $\mu$m. Thymidine utilisation will occur only up to a depth short of this zero point. The rationale behind using hyperbaric oxygen is therefore to increase the depth of [$^3$H]dT utilisation and hopefully to overcome the problems experienced by Johnson & Bond (1961) and Lieb & Lisco (1966) where uptake of label was only found in the superficial cells of incubated tissue fragments. Fabrikant *et al.* (1969) claimed that maximal depth of labelling could be achieved using an oxygen pressure of 3 atmospheres; no improvement on labelling beyond 500 $\mu$m into the tissue could be obtained by raising the oxygen pressure above 3 atmospheres. Meyer & Connor (1977) claimed to confirm this.

Using hyperbaric oxygen at a pressure of 3 atmospheres, we only achieved a rim of labelling around the edges of lung tumour fragments. The rim effect appears to be the result of a metabolic gradient of oxygen tension and diffusing metabolites, where any change in these latter components will affect the final rim depth. The effect of changing oxygen tension has already been mentioned and our early trials with the technique confirmed deeper labelling when using hyperbaric oxygen compared with 95% $O_2$/5% $CO_2$ at atmospheric pressure. In some cases we incubated tumour fragments with [$^3$H]-uridine and labelling occurred throughout all the fragments though a decrease in labelling intensity towards their centre was seen (data not shown). This suggests that the metabolites do in fact diffuse throughout the tissue fragments.

The availability of sufficient oxygen may determine the degree of utilisation. Thus, tumour cells utilising [$^3$H]-uridine require less oxygen than the S-phase tumour cells to take up and incorporate exogenous [$^3$H]dT. For a given oxygen concentration and metabolite the rim depth probably also depends on the particular cell type in the tissue under study. We noted on several occasions where stromal fibroblast labelling was prominent in solid tumours that within the same tumours and in identical fields counted, the $D_{70}$ for fibroblasts was considerably greater than that for tumour cells. We also noted, during some preliminary labelling studies using foetal rat liver, that the $D_{70}$ for more actively proliferating haemopoietic cells was much less than that of the hepatocytes.

Our rim of [$^3$H]dT labelling varied in depth from tumour to tumour and the $D_{70}$ values calculated suggested that the depth of labelling is inversely proportional to the TLI and these 2 factors may be exponentially related. A relationship between TLI and depth has also been observed by Meyer & Connor (1977) who referred to a steeper labelling gradient in highly cellular tissues with high TLI when compared with tissues of low cellularity and TLI. The same workers noted a lower TLI in a deeper zone of the tumour when compared with the periphery. Chavaudra *et al.* (1979) also discuss the variation of TLI with depth in labelling in tissue fragments stating that in some squamous carcinomata labelling is maximal between 21–40 $\mu$m and in others between 80–100 $\mu$m deep.

The rim effect and its variability has further implications concerning this type of work. Firstly, it raises questions concerning experiments where tissue is homogenised after incubation and the radioactivity present detected by scintillation counting. As labelling depends on the precursor used, and since differences in TLI and $D_{70}$ values do exist between different cell populations in the same tissue, the validity of such work (Sky-Peck, 1971) must be in doubt. In the light of our experience with lung tumours, even when considering a single histological tumour group, one cannot assume that tumour cells are consistent in their behaviour under these experimental conditions from case to case.

Secondly, the choice of which areas to consider for counting TLI often seems quite arbitrary. Meyer & Connor (1977), Chavaudra *et al.* (1979) and others have commented that much of the current data available on *in vitro* human tumour [$^3$H]dT labelling may underestimate tumour cell TLIs since areas deep in the fragments with suboptimal labelling are being counted. Hainau *et al.* (1977) would score tumour cells in a field where no labelled tumour cells were present if labelled stroma was seen, this being taken as evidence of adequate conditions for tumour cell labelling. What has been presented and discussed above suggests that this is not necessarily so. In our tumours with a low labelling frequency, knowing the depth to which labelling occurred we could assume adequate conditions for counting along the entire fragment edge even if the edge only contained one labelled cell; one cannot omit counting peripheral zones like these just because so few labelled cells are present as this would falsely raise the TLI.

In some of our cases we questioned whether a low TLI reflected technical difficulties involving uptake of [$^3$H]dT. However, the MI values do not support this idea as the proportion of TLI:MI is fairly constant with low values of TLI having proportionally low values of MI. We feel our method of "tailoring" as far as practicable, the depth of our rim for counting from labelling patterns on histograms for each tumour and then counting *all* areas in that rim in each fragment edge considered, overcomes many of these doubts and gives optimum results for tumour cell TLI in the fragment areas counted.

Data on human tumour cell kinetics is of interest since it reflects one of the factors contributing directly to the growth of the neoplasm. Such data can be studied in conjunction with the *in vivo* growth of the tumour in the patient.

Comparisons such as these depend on the fact that the *in vitro* techniques of [$^3$H]dT labelling are reproducible and adequately reflect the *in vivo* situation. Direct comparison of methodology has been made by Johnson & Bond (1961) and Denekamp & Kallman (1973) all of whom conclude that *in vitro* systems can and do reflect the *in vivo* state. This point is further discussed by Tubiana (1971) and Steel (1977) with similar conclusions. In particular, doubts may be raised concerning the effects of transporting tumour tissue at 4°C prior to incubation at 37°C. Several groups have investigated the effect of this cold shock with direct reference to [$^3$H]dT labelling of tumours. Fabrikant *et al.* (1969) and Steel & Bensted (1965) both found that thymidine uptake was unchanged during storage of tissue at 4°C for up to 6 h, when compared with tissue processed immediately. Mayer & Bauer (1975) found that even storing tissue at room temperature for up to 135 min made no difference to labelling.

We hope it will also be possible, at a suitable point in the future to follow-up the 57 patients and determine whether the TLI has any relationship to clinical progress taking into account histological cell type and stage.

This work was supported by a grant from the National Coal Board and from the Scottish Hospital Endowment Research Trust to Dr D. Lamb. We thank: Mr R. McCormack, Mr P. Walbaum and Mr E. Cameron, Consultant Surgeons at the Thoracic Unit, City Hospital, Edinburgh, their colleagues, and the nursing and theatre staff for the supply of fresh lung resection specimens; Mr R. Hogg, Mr A. Smith and Mr S. McKenzie for technical assistance; Mrs Fiona Govan for typing this manuscript.

**References**

CHAVAUDRA, N., RICHARD, J.M. & MALAISE, E.P. (1979). Labelling index of human squamous cell carcinomas. Comparison of *in vivo* and *in vitro* labelling methods. *Cell Tissue Kinet.*, **12**, 145.

CLEAVER, J.E. (1967). *Thymidine metabolism and cell kinetics*. Amsterdam: North Holland. p. 00.

COONS, H., NORMAN, A. & NAHUM, A.M. (1966). *In vitro* measurements of human tumour growth. *Cancer*, **19**, 1200.

DENEKAMP, J. & KALLMAN, R.F. (1973). *In vitro* and *in vivo* labelling of animal tumours with tritiated thymidine. *Cell Tissue Kinet.*, **6**, 217.

FABRIKANT, J.I. & WISSEMAN, C.L. (1968). *In vitro* incorporation of tritiated thymidine in normal and neoplastic tissues. *Radiology*, **90**, 361.

FABRIKANT, J.I., WISSEMAN, C.L. & VITAK, M.J. (1969). The kinetics of cellular proliferation in normal and malignant tissues. II. An *in vitro* method for incorporation of tritiated thymidine in human tissues. *Radiology*, **92**, 1309.

HAINAU, B., DOMBERNOWSKY, P., HANSEN, H.H. & BORGESKOV, S. (1977). Cell proliferation and histologic classification of bronchogenic carcinoma. *J. Natl Cancer Inst.*, **59**, 1113.

JOHNSON, H.A. & BOND, V.P. (1961). A method of labelling tissues with tritiated thymidine *in vitro* and its use in comparing rates of cell proliferation in duct epithelium, fibroadenoma, and carcinoma of human breast. *Cancer*, **14**, 639.

LIEB, L.M. & LISCO, H. (1966). *In vitro* uptake of tritiated thymidine by carcinoma of the human colon. *Cancer Res.*, **26**, 733.

LIVINGSTON, R.B., AMBUS, U., GEORGE, S.L., FREIREICH, E.J. & HART, J.S. (1974). *In vitro* determination of thymidine-$^3$H labelling index in human solid tumours. *Cancer Res.*, **34**, 1976.

MALAISE, E.P., CHAVAUDRA, N. & TUBIANA, M. (1973). The relationship between growth rate, labelling index and histological type of human solid tumours. *Eur. J. Cancer*, **9**, 305.

MATTHEWS, M.J. (1976). Problems in morphology and behaviour of bronchopulmonary malignant disease. In *Lung Cancer: Natural History, Prognosis and Therapy* (Eds. Israel & Chahinian.) New York: Academic Press.

MEYER, J.S. & BAUER, W.C. (1975). *In vitro* determination of tritiated thymidine labelling index (LI). Evaluation of a method utilizing hyperbaric oxygen and observations on the LI of human mammary carcinoma. *Cancer*, **36**, 1374.

MEYER, J.S. & CONNOR, R.E. (1977). *In vitro* labelling of solid tissues with tritiated thymidine for autoradiographic detection of S-phase nuclei. *Stain Technol.*, **52**, 185.

MUGGIA, F.M. (1973). Correlation of histologic types with cell kinetic studies in lung cancer. *Cancer Chemother. Rep.*, **4**, 69.

NORDENSKJÖLD, B., ZETTERBERG, A. & LÖWHAGEN, T. (1974). Measurement of DNA synthesis by $^3$H-thymidine incorporation into needle aspirates from human tumours. *Acta Cytol.*, **18**, 215.

SKLAREW, R.J., HOFFMAN, J. & POST, J. (1977). A rapid *in vitro* method for measuring cell proliferation in human breast cancer. *Cancer*, **40**, 2299.

SKY-PECK, H.H. (1971). Effects of chemotherapy on the incorporation of $^3$H-thymidine into DNA of human neoplastic tissue. *Natl Cancer Inst. Monogr.*, **34**, 197.

STEEL, G.G. (1977). Growth kinetics of tumours. In *Cell Population Kinetics in Relation to the Growth and Treatment of Cancer*. Oxford: University Press. Chapter 3, p. 106.

STEEL, G.G. & BENSTED, J.P.M. (1965). *In vitro* studies of cell proliferation in tumours. I. Critical appraisal of methods and theoretical considerations. *Eur J. Cancer*, **1**, 275.

STRAUS, M.J. & MORAN, R.E. (1977). Cell cycle parameters in human solid tumours. *Cancer*, **40**, 1453.

TERZ, J.J., CURUTCHET, H.P. & LAWRENCE, W.Jr. (1971). Analysis of the cell kinetics of human solid tumors. *Cancer*, **28**, 1100.

TITUS, J.L. & SHORTER, R.G. (1965). Labelling of human tumors with tritiated thymidine. *Arch. Pathol.*, **79**, 324.

TUBIANA, M. (1971). The kinetics of tumour cell proliferation and radiotherapy. *Br. J. Radiol.*, **44**, 325.

TUBIANA, M. & MALAISE, E.P. (1976). Growth rate and cell kinetics in human tumours: some prognostic and therapeutic implications. In *Scientific Foundations of Oncology*. (Eds. Symington & Cancer) London: Heinemann. p. 126.

WOLBERG, W.H. & BROWN, R.R. (1962). Autoradiographic studies of *in vitro* incorporation of uridine and thymidine by human tumour tissue. *Cancer Res.*, **22**, 1113.